# Clinical Advances in the Diagnosis and Treatment of Cerebrovascular Diseases

**DOI:** 10.3390/jcm14010198

**Published:** 2025-01-01

**Authors:** Asad Lak, Mario Zanaty

**Affiliations:** Department of Neurosurgery, University of Iowa, Iowa City, IA 52242-1181, USA; asad-lak@uiowa.edu

The management of cerebrovascular diseases has significantly evolved over the last decade or so [1,2], and technological advancements resulting in safer, more effective and less invasive approaches have dramatically changed the outlook for these patients [3,4,5,6]. Traditionally treated with invasive surgical procedures with a high risk of morbidity and mortality, the recent years have seen a paradigm shift with endovascular therapy taking over treatment for intracranial vascular lesions [1,3]. While endovascular approaches are safer and equally as effective as open surgical approaches, there arises a question about the comparability of different approaches and optimum patient selection for each approach [7,8]. Moreover, with the emergence of big data and artificial intelligence in healthcare and the availability of newer endovascular devices, lesions which were previously considered untreatable are now amenable to endovascular treatment [9,10,11,12]. Hence, it is imperative to critically evaluate these rapid advancements in the management of cerebrovascular diseases in order to push the field forwards in a safer and effective manner.

This Editorial refers to the Special Issue “*Clinical Advances in Diagnosis and Treatment of Cerebrovascular Diseases*”. This Special Issue attempts to identify promising advancements in the diagnosis and management of cerebrovascular diseases, focusing on new imaging techniques, treatment devices and markers for functional outcomes. The goal of this Special Issue is to summarize the exciting advancements in the field of cerebrovascular disease and identify areas for future research.

A total of five manuscripts were submitted for publication and, after rigorous peer review, were selected for publication. The manuscripts accepted for publication are as follows:

1. The Relative Cerebral Blood Volume (rCBV) < 42% is Independently Associated with Collateral Status in Anterior Circulation Large Vessel Occlusion by Lakhani et al.
2. Flow Diversion for Endovascular Treatment of Intracranial Aneurysms: Past, Present and Future Directions by Gaub et al.
3. Contemporary Methods for Detection and Intervention of Distal Medium and Small Vessel Occlusions by Piscopo et al.
4. Association between Left Atrial Appendage Morphology and Clot Histology in Patients with Embolic Ischemic Stroke: An Exploratory Study by Lengvenis et al.
5. Assessment of Blood Loss During Neuroendovascular Procedures by Goutnik et al.

The submitted manuscripts evaluated several different aspects of the management of cerebrovascular disease. The areas of significance ranged from utilizing currently available imaging parameters to predict functional outcomes following endovascular treatment in large vessel occlusion; using advanced imaging techniques for improved diagnoses; evaluating the potential of mechanical thrombectomy in distal medium and small vessel occlusions; and deconvoluting the role of atrial appendage morphology in embolic stroke and the role of flow diverters in the treatment of intracranial aneurysms.

Manuscript 1 analyzed the utility of relative cerebral blood volume (rCBV) as a marker for functional outcomes in patients with anterior circulation large vessel occlusion (LVO). The authors hypothesized that rCBV < 42% is a marker for poor collateral status and hence predicts poor outcomes. They demonstrated that the rCBV can be easily calculated from a pre-treatment cerebral perfusion scan. The authors’ results reveal that rCBV is independently associated with poor collateral status and hence can be used as a functional marker of outcomes.

Manuscript 2 took us on a journey of the development of flow diverters and parent vessel remodeling as a method of treatment in intracranial aneurysms. The manuscript summarizes the key landmark trials resulting in the approval of flow diverter devices including PITA, PUFS and PREMIER. It also provides a natural history of flow diverter devices from PED to WEB and FRED. The authors highlight the current indications for the use of flow diverters, including unruptured aneurysms, particularly those with a wide neck. They also highlight the challenges of and strategies for utilizing flow diverters in ruptured intracranial aneurysms. Moreover, the authors point towards future directions in the field of flow diversion including bioresorbable flow diverters, the use of transcriptomics to study flow diversion-related changes and incorporating machine learning techniques to precisely predict aneurysm occlusion following treatment.

Manuscript 3 focused on endovascular thrombectomy for distal medium- and small-vessel occlusion. The authors summarized the current literature on endovascular treatment of distal medium- and small-vessel occlusions. They provided future directions for refined imaging technologies like multiphase CTA which may assist in detecting these small occlusions. The authors also advocate for the incorporation of artificial intelligence for the refined processing of pre-treatment imaging. The authors identified the lack of clinical trials evaluating outcomes following treatment in distal medium- and small-vessel occlusions and suggest larger clinical trials to determine the safety and efficacy of this approach.

Manuscript 4 evaluated the relationship between different morphologies of left atrial appendage (LAA) and clot composition in large-vessel occlusion. They demonstrated low fibrin composition in patients with chicken wing-type LAA. The authors suggest that differences in clot composition among different LAA morphology subtypes may provide direction for workup and secondary prevention in patients with embolic stroke of unknown etiology (ESUS). A better understanding of flow dynamics in patients with different LAA morphology may help identify strategies for secondary prevention in patients with ESUS.

Manuscript 5 quantified the amount of blood loss in different endovascular procedures. The authors’ results verified minimal blood loss during these procedures and highlighted that advanced age and pre-operative antiplatelet/anticoagulant use increases the risk of bleeding.

The research articles submitted to this Special Issue excellently summarize the upcoming advancements in the field of cerebrovascular diseases. These manuscripts also highlighted gaps and directions for future research.

- Mechanical thrombectomy has dramatically changed the outlook for patients with large-vessel occlusion [2,4,5,6]. Assessment of a patient’s collateral circulation status pre-operatively will be helpful in identifying high-risk patients particularly as the indications for thrombectomy are expanding. Additionally, larger studies are needed to determine if collateral status can be predicted from a pre-operative CT perfusion scan.
- The emergence of flow diverters has changed the management of intracranial aneurysms from focusing on aneurysm occlusion to parent-vessel remodeling. Future studies should highlight the role of flow diverters in the management of ruptured and small intracranial aneurysms. Moreover, newer devices, including bioresorbable flow diverters, and the incorporation of artificial intelligence/machine learning to accurately predict outcomes are needed.
- The current strategy for the management of distal medium- and small-vessel occlusion is conservative management. Future studies should focus on imaging techniques to identify these occlusions and also provide information regarding the safety and efficacy of treatment with newer microcatheters for these patients.
- Approximately half of patients with stroke have unidentified etiology, commonly referred to as ESUS. Assessment of the left atrial appendage morphology and flow dynamics studies might provide important information for clot development and propagation in these patients. The identification of differences in flow dynamics among different atrial appendage morphologies may lead to improved secondary prevention strategies.
